# Epidemiological characteristics of human psittacosis in Guangdong Province, China, 2019–2024

**DOI:** 10.3389/fpubh.2025.1651380

**Published:** 2025-10-20

**Authors:** Yali Zhuang, Yu Wang, Shujun Zhang, Chengcong Li, Ting Hu, Chenghuan Zhang, Zenglong Huang, Qiuhong Zeng, Ze Liu, Canhui Weng, Lixian Luo, Mingqi Zou, Jing Zeng, Wenting Guo, Wenyan Li, Min Kang, Aiping Deng

**Affiliations:** ^1^Institute of Infectious Disease Control and Prevention, Guangdong Provincial Center for Disease Control and Prevention, Guangzhou, China; ^2^Institute of Infectious Disease Control and Prevention, Kaiping Center for Disease Control and Prevention, Jiangmen, China; ^3^Institute of Infectious Disease Control and Prevention, Maoming Municipal Center for Disease Control and Prevention, Maoming, China; ^4^Department of Infectious Disease Control and Prevention, Yantian District Center for Disease Control and Prevention, Shenzhen, China; ^5^Institute of Infectious Disease Control and Prevention, Qujiang Center for Disease Control and Prevention, Shaoguan, China; ^6^Institute of Infectious Disease Control and Prevention, Heyuan Center for Disease Control and Prevention, Heyuan, China; ^7^Disease Control Unit, Chaoan Center for Disease Control and Prevention, Chaozhou, China; ^8^Institute of Infectious Disease Control and Prevention, Daya Bay Economic and Technological Development Zone Center for Disease Control and Prevention, Huizhou, China; ^9^Disease Control Unit, Chaonan Center for Disease Control and Prevention, Shantou, China; ^10^Institute of Infectious Disease Control and Prevention, Meizhou Municipal Center for Disease Control and Prevention, Meizhou, China

**Keywords:** psittacosis, epidemiological characteristics, poultry, underlying diseases, retrospective survey

## Abstract

**Background:**

Psittacosis is a non-statutory infectious disease and receives relatively low attention in China. Since 2019, the incidence of psittacosis in Guangdong Province has been continuously increasing. Therefore, it is necessary to understand the epidemiological characteristics, providing a basis for optimizing psittacosis prevention and control.

**Methods:**

This study included psittacosis cases reported in Guangdong Province from 2019 to 2024. Data were collected and a retrospective survey was conducted. The spatiotemporal distribution, clinical manifestations and epidemiological exposure histories were analyzed. Logistic regression model was used to explored the risk factors for psittacosis pneumonia.

**Results:**

A total of 435 psittacosis cases were reported in Guangdong Province. It demonstrated an overall increasing trend in the incidence rate, with cases predominantly occurring in winter and spring. Nansha District in Guangzhou (*p* < 0.001), Boluo in Huizhou (*p* < 0.001) and Shunde in Foshan (*p* = 0.001) were identified as hotspots for psittacosis. The incidence rate of psittacosis was higher in males (χ^2^ = 17.26, *p* < 0.001) and in the 50–79 age group (χ^2^ = 123.45, *p* < 0.001). Univariate regression analysis showed that underlying diseases are a risk factor for psittacosis pneumonia [OR (95% CI) = 2.47(1.42, 3.31), *p* = 0.01]. There were 162 cases with a history of epidemiological exposure, but only 42 cases (25.93%) used protective measures.

**Conclusion:**

The incidence of psittacosis has been increasing in Guangdong Province recently, posing a threat to individuals with poultry exposure. In the future, it is suggested to enhance the monitoring of individuals with daily contact with poultry, particularly for the older adult, in winter and spring.

## 1 Introduction

Psittacosis is a natural focal disease caused by *Chlamydia psittaci* in humans, birds, and some mammals (1). Human infection occurs through broken skin, mucous membranes, and digestive tract (2). Furthermore, there have been instances of human-to-human transmission of psittacosis (3, 4).

Human psittacosis presents with a wide spectrum of clinical manifestations, ranging from asymptomatic infection to severe systemic illness. The most common presentation is an atypical pneumonia with symptoms including fever, headache, myalgia, and dry cough, which may progress to severe respiratory complications if left untreated (5–7). Extrapulmonary manifestations can include hepatitis, myocarditis, encephalitis, and endocarditis, making diagnosis challenging due to its resemblance to other respiratory pathogens (8, 9). The incubation period typically ranges from 5 to 14 days, though it can extend up to 4 weeks in some cases (1, 10). Early recognition and appropriate antibiotic treatment are crucial, as untreated psittacosis can result in mortality rates of 15%−20%, while timely treatment reduces this to less than 1% (3).

From a public health perspective, psittacosis poses significant challenges due to its zoonotic nature, potential for outbreaks in occupational settings, and diagnostic difficulties. Occupational groups at higher risk include poultry workers, veterinarians, pet shop employees, and laboratory personnel working with birds (1, 11). The disease has been associated with nosocomial transmission and community outbreaks linked to infected birds in public spaces (12, 13). Furthermore, the non-specific clinical presentation often leads to misdiagnosis and delayed treatment, potentially contributing to antibiotic resistance and prolonged infectious periods. The economic burden includes not only direct healthcare costs but also productivity losses due to prolonged illness and outbreak investigations (14).

The disease burden caused by psittacosis in the Netherlands was estimated to be 222 DALYs per year (95% CI 172–280) over the period 2012–2014 (15). A multicenter observational study showed that psittacosis accounted for approximately 2.1% of complicated or atypical pulmonary infection in China from 2019 to 2021(16). However, psittacosis is not a legally notifiable infectious disease in most parts of China currently. Apart from a few provinces such as Jiangsu, which has established regulations for managing psittacosis and classified it as a Category C infectious disease, other provinces do not mandatorily require the reporting of psittacosis cases. Research on psittacosis primarily consists of clinical case reports, with fewer studies focusing on epidemiology. There is limited research on the prevalence and disease burden of psittacosis. This study analyzes the epidemiological characteristics of psittacosis cases reported through the network reporting system in Guangdong Province from 2019 to 2024. This study aims to understand the distribution of psittacosis, providing evidence for the further optimization of prevention and control measures against the disease.

## 2 Materials and methods

### 2.1 Data source

This study is a retrospective study. The subjects consisted of 435 cases of psittacosis collected in Guangdong Province from January 2019 to December 2024 through the National Notifiable Disease Reporting System (NNDRS, https://10.249.6.18:8881/cdc/, authorization for access) using two search strategy: (1) identification of cases reported as “other infectious diseases” with “*psittacosis*” specified in the supplementary information field, and (2) identification of cases reported as “other diseases” without supplementary explanations but containing *psittacosis*-related testing information in the case report remarks. Psittacosis cases which reported in NNDRS as other infectious disease but specifically noted as psittacosis in the remarks, are used as the subjects for this investigation, since psittacosis is currently not a notifiable infectious disease in China.

All included psittacosis cases were laboratory confirmed: 271 cases were confirmed by Next Generation Sequencing testing conducted at hospitals; 67 cases were confirmed by nucleic acid testing performed at Centers for Disease Control and Prevention; 1 case was confirmed by psittacosis antigen detection; 96 cases had confirmed laboratory results but the specific testing methods could not be determined from the available records. A retrospective survey was conducted on the cases using the national telephone epidemiological investigation system. The survey primarily included basic information about the clinical manifestations and poultry exposure situations. Demographic data were sourced from the China Disease Prevention and Control Information System. The annual incidence rate was calculated using the total population of Guangdong Province each year.

### 2.2 Statistical methods

The chi-square test, multivariate logistic regression and wavelet analysis were performed using R software (version 4.4.1,). In brief, wavelet analysis is used to analyze the periodicity of psittacosis, employing the “WaveletComp” package (version 1.1). The number of psittacosis cases were collected from 21 cities of Guangdong Province from January 2019 to December 2024. Wavelet analysis identified coherent periodic patterns and phase relationships between psittacosis cases and these external variables. A local periodic function (the wavelet) to decompose fluctuations of time series was adopted to observe during a small time interval into a series of different periodicity. The importance of periodicity (wavelet power) was then plotted in contour plots as a function of time. The chi-square test was applied to compare gender differences in psittacosis incidence. Multivariate logistic regression was used to explore the association between psittacosis pneumonia with gender, age, area, underlying diseases, and weekly contact frequency. Two-sided tests were conducted with a significance level of α = 0.05. Then, the periodicity and the time of the fluctuations can both be determined. Trends in incidence rates over time were determined by using joinpoint regression models using SEER's Joinpoint Regression Program (version 3.4.3). ArcMap 10.2 software was used for mapping and hotspot analysis, employing the Getis-OrdGi tool to analyze regional hotspots of psittacosis in Guangdong Province. All software tools used in this study were legally licensed.

## 3 Results

### 3.1 Temporal distribution

**Figure 1:**
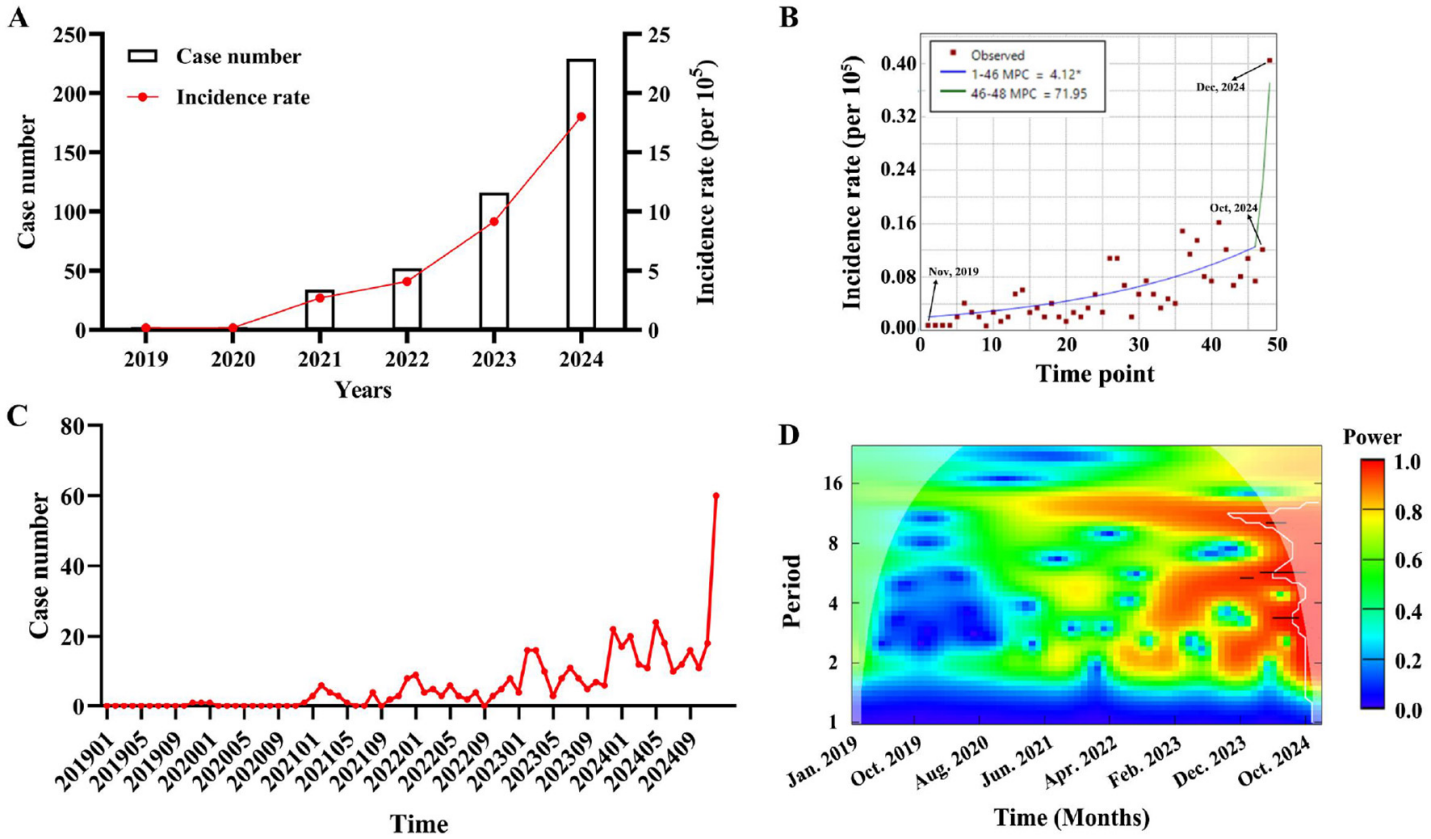
The temporal dynamics of psittacosis cases in Guangdong Province from 2019 to 2024. **(A)** Annual number of reported cases and incidence rate of psittacosis were analyzed. **(B)** Monthly percentage change of psittacosis was calculated using Joinpoint regression. **(C)** Monthly distribution of psittacosis cases from January 2019 to December 2024. **(D)** Contour plot of the real part of wavelet coefficients for psittacosis cases over time series. Colors from blue to red represent increasing power.

From 2019 to 2024, the total number of reported cases of psittacosis in Guangdong Province was 435, which was 2, 2, 34, 52, 116 and 229, respectively. The incidence rate is also increasing year by year (Figure 1A). Joinpoint regression analysis was employed to evaluate temporal trends in psittacosis incidence. The results revealed a significant overall upward trend, with an average monthly percentage change (MPC) of 4.12% from Jan, 2019 to Oct, 2024. Notably, a sharp acceleration in incidence occurred from October to December 2024, with an MPC of 71.95%, suggesting a potential outbreak or enhanced surveillance during this period (Figure 1B). To explore the periodicity of psittacosis incidence, the number of cases per month was collected (Figure 1C), and wavelet analysis was used to study the incidence cycle. From Jan, 2023 to Dec, 2024, cycles of 4–6 months exhibited higher power, indicating significant periodic fluctuations in psittacosis during this period. Cycles of 10–14 months exhibited high power, indicating significant annual periodic fluctuations during this period from Jun, 2021 to Dec, 2024 (Figure 1D). It is speculated that psittacosis has a high incidence in winter and spring.

### 3.2 Regional distribution

**Figure 2:**
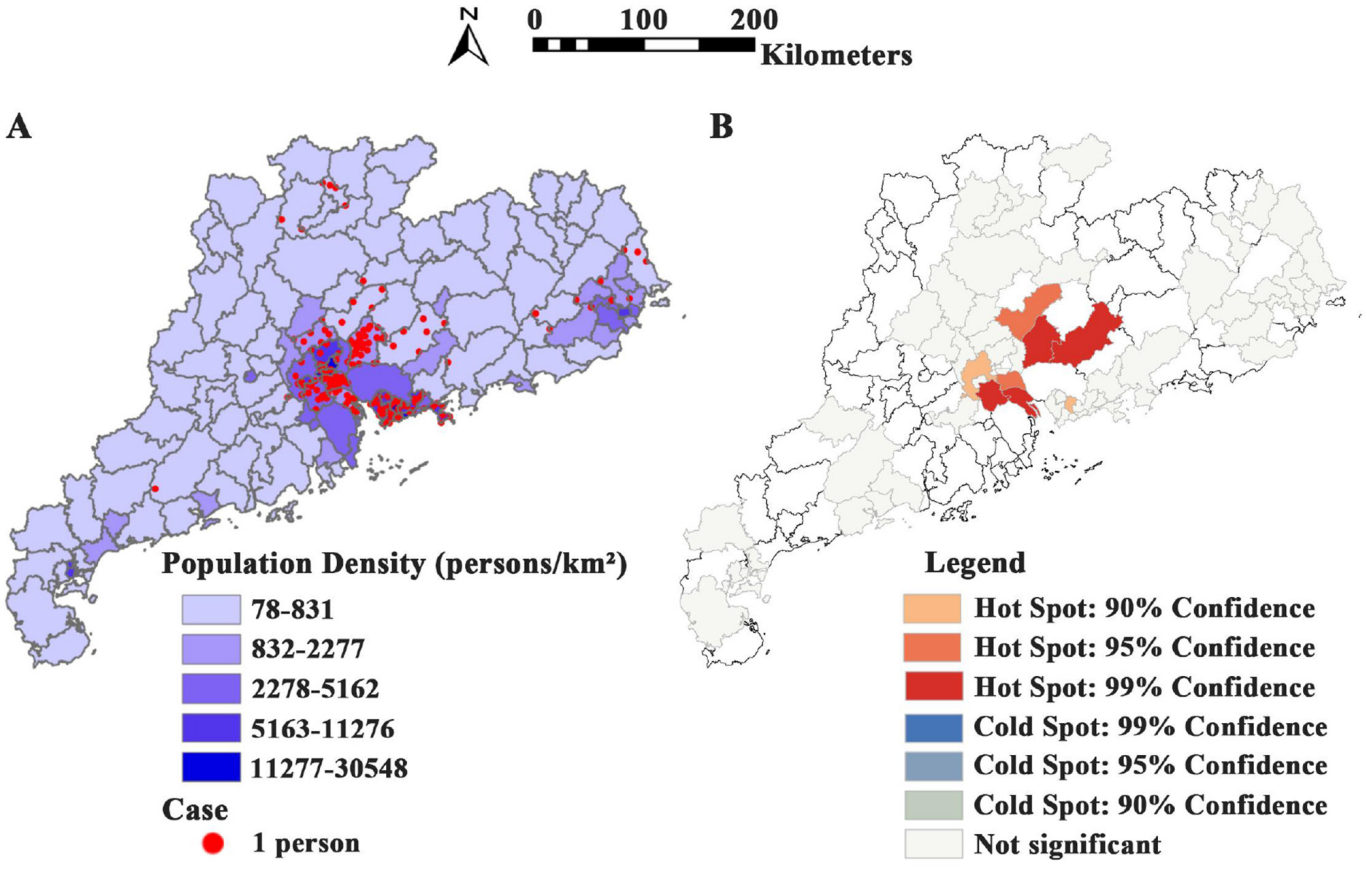
Spatial distribution of psittacosis cases in Guangdong Province from 2019 to 2024. **(A)** Population density and case distribution in Guangdong Province from 2019 to 2024. **(B)** Hotspot map showing cumulative distribution of psittacosis cases across cities in Guangdong Province from 2019 to 2024.

Population density and case distribution in Guangdong Province from Jan. 2019 to Dec. 2024 (Figure 2A). A total of 435 psittacosis cases in 17 cities in Guangdong Province were reported to the NNDRS. In 2019, only one city in Guangdong Province, Shenzhen, reported psittacosis, and two cities reported cases (Shenzhen and Shaoguan) in 2020. From 2021 to 2024, the number of cities reporting cases increased to 9, 9, 13, and 27 respectively. Since 2019, most cases have been reported in Guangzhou (42.99%) and Shenzhen (23.68%). The Getis-Ord-G hotspot analysis revealed that Nansha District in Guangzhou (*p* < 0.001), Boluo in Huizhou (*p* < 0.001), and Shunde District in Foshan (*p* = 0.001) were high-incidence areas for psittacosis (Figure 2B).

### 3.3 Population distribution

**Figure 3:**
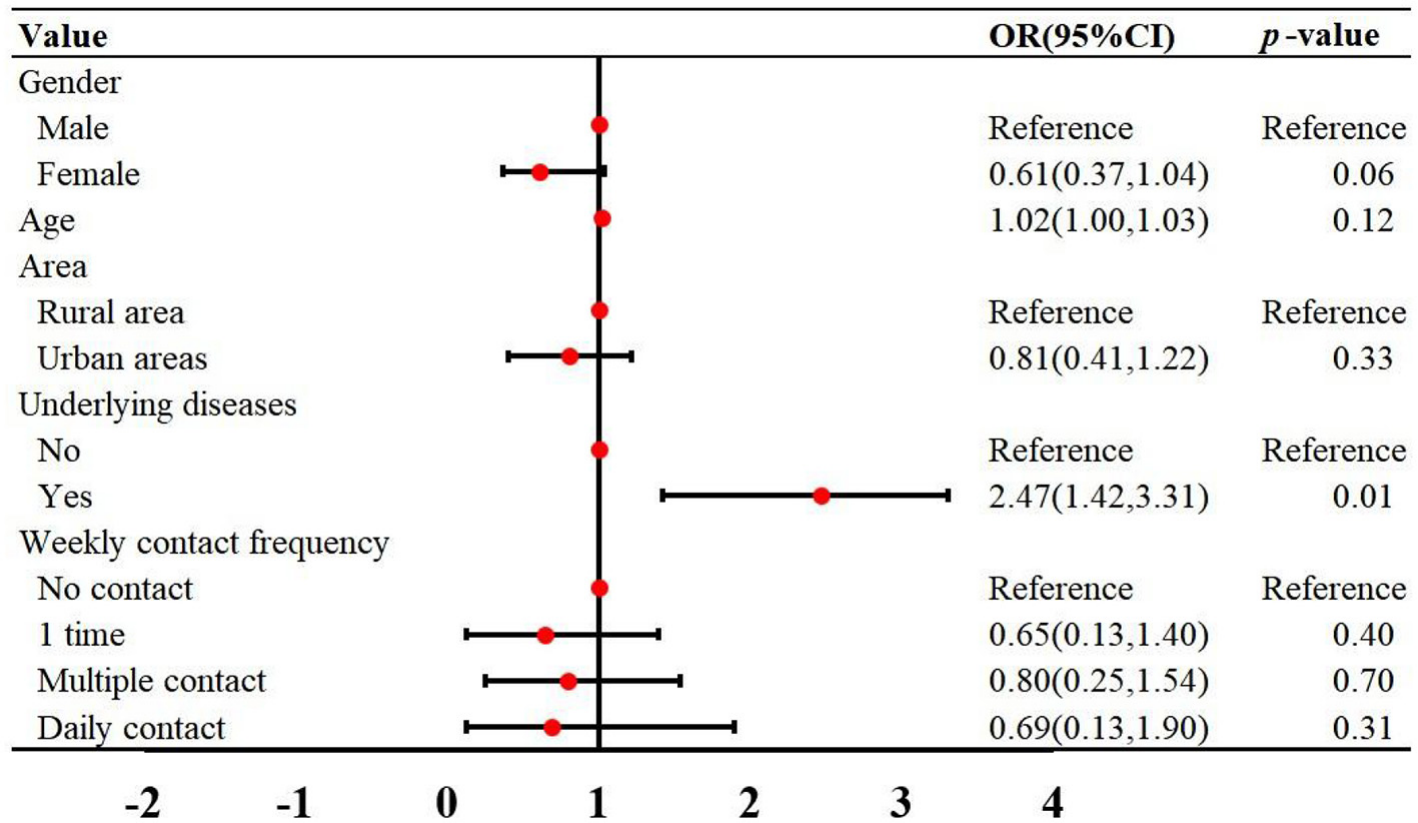
Multivariate logistic regression was used to analyze the risk factors for psittacosis pneumonia in Guangdong Province from 2019 to 2024. OR, odds ratios; CI, confidence interval.

The demographic characteristics of the reported cases were shown in Table 1. Among the 435 cases, there were 261 male and 174 female, with a male-to-female ratio of 1:0.67. The incidence rate in males was higher than in females (χ^2^ = 17.26, *p* < 0.001) (Table 1). The ages of the cases ranged from 3 to 93 years old, with a median age of 58. The incidence rates in the 50–60 and 60–70 age groups were higher than those in other age groups (Table 1). Of the 162 cases successfully follow-up, 46 (28.39%) had underlying diseases, 109 (64.28%) did not, and 7 (4.32%) were unclear. The most common symptoms were fever (95.06%) and pneumonia (30.25%) (Table 2). Multivariate regression analysis showed that underlying diseases are positive associated with psittacosis pneumonia [OR (95% CI) = 2.47 (1.42, 3.31)]. Compared to cases without psittacosis pneumonia, patients with underlying conditions are more likely to develop complications such as pneumonia after being infected with the psittacosis pathogen (Figure 3).

**Table 1:** Basic characteristics of psittacosis cases in Guangdong Province from 2019 to 2024.

**Characteristics**	**Number of cases (%)**	**Incidence rate (per 10^5^)**	**χ^2^**	***p*-value**
**Age**				
< 10	2 (0.46)	0.16^a^	123.45	< 0.001
[10–20)	1 (0.23)	0.08^a^		
[20–30)	12 (2.76)	0.98^a^		
[30–40)	37 (8.51)	3.02^a^		
[40–50)	62 (14.25)	5.06^a^		
[50–60)	139 (31.95)	11.34^a^		
[60–70)	109 (25.06)	8.90^b^		
[70–80)	61 (14.02)	4.98^b^		
≥80	12 (2.76)	0.98^a^		
**Gender**				
Male	261 (60.0)	21.3	17.26	< 0.001
Female	174 (40.0)	14.2		

Categorical variables were presented as number (percentage). Chi-square test was used to compare differences between groups. *p*-value < 0.05 was considered statistically significant. Bonferroni method was used for pairwise comparisons, and different letters (a or b) indicate statistically significant differences.

**Table 2:** Underlying diseases and clinical symptoms of participants.

**Category**	**Number of cases (%)**
**Underlying Diseases**	
Hypertension	18 (11.11)
Diabetes	13 (8.02)
Emphysema	4 (2.47)
Hyperlipidemia	2 (1.23)
Coronary heart disease	3 (1.85)
High blood glucose	5 (3.09)
Cancer	1 (0.62)
No underlying disease	109 (64.28)
Unclear	7 (4.32)
**Clinical symptoms**	
Fever	154 (95.06)
Pneumonia	49 (30.25)
Dry cough	38 (23.46)
Productive cough	42 (25.93)
Headache	31 (19.14)
Fatigue	32 (19.75)
Chills	19 (11.73)
Poor appetite	14 (8.64)
Dyspnea	13 (8.02)
Muscle pain	13 (8.02)

Individual presented with multiple underlying diseases and clinical symptoms.

### 3.4 Epidemiological exposure of the cases

Among the 162 cases, 108 cases (66.67%) had a history of bird epidemiological exposure, while the remaining 54 cases (33.33%) did not. An analysis of the 108 cases with poultry exposure revealed that the most common exposure animals were chickens (30.86%), parrots (18.52%), and ducks (19.14%). The most common sources of poultry exposure were food market (38.89%) and bird markets (33.33%). The most common exposure methods were breeding (51.23%), farming (29.63%), and cleaning coops (25.92%). Notably, 77.78% of the cases did not use protective measures when in contact with poultry. Only 29 cases (17.90%) reported using protective measures, 20 cases simultaneously wearing masks and rubber gloves, while 22 cases only wear rubber gloves (Table 3).

**Table 3:** Poultry exposure in the 45 days to illness onset in surveyed cases.

**Exposure characteristics**	**Number of cases (%)**
**Exposure to birds** ^#^	
Chickens	50 (30.86)
Parrots	30 (18.52)
Ducks	31 (19.14)
Doves	13 (8.02)
Goose	14 (8.64)
None	54 (33.33)
**Source of the birds** ^#^	
Food market	63 (38.89)
Bird market	54 (33.33)
Buy online	16 (9.88)
Other Sources	35 (21.6)
Unclear	48 (29.63)
**Frequency of wearing protective equipment**	
Never	126 (77.78)
Sometimes	20 (12.34)
All times	9 (5.56)
Unclear	7 (4.32)
**Exposure way** ^#^	
Breeding	83 (51.23)
Farming	48 (29.63)
Cleaning coops	42 (25.92)
Purchase live poultry	28 (17.28)
Slaughter	28 (17.28)
**The frequency of contact**	
1 time	16 (9.88)
2–14 time	14 (8.64)
15–29 time	7 (4.32)
30–44 time	8 (4.94)
Every day	117 (72.22)
**Personal protective equipment**	
Masks and rubber gloves	20 (12.35)
Only with rubber gloves	22 (13.58)
None	120 (74.07)

Categorical variables were presented as number (percentage). ^#^Multiple-choice questions.

## 4 Discussion

The number of psittacosis cases in Guangdong Province has been steadily increasing year by year since 2021, with the cases from 2022 to 2024 accounting for 91.26% of the total cases in the past 6 years. This trend may be attributed to multiple factors beyond the widespread clinical application of metagenomic sequencing leading to an increase in the detection rate of pathogens (3, 17). First, enhanced surveillance capabilities and increased attention from disease control departments have contributed to improved case detection (18). Second, the COVID-19 pandemic has accelerated the adoption of advanced diagnostic technologies, particularly metagenomic next-generation sequencing (mNGS), which has significantly improved the diagnostic capacity for atypical pneumonia pathogens including *C. psittaci* (19, 20). Studies have demonstrated that mNGS can increase pathogen identification rates from 40.8% (using PCR) to 74.2% in severe community-acquired pneumonia cases (21). Third, the post-COVID-19 era has led to heightened awareness among clinicians regarding respiratory infections of unknown etiology, potentially resulting in more comprehensive diagnostic workups (17).

A multicenter observational study indicated that psittacosis mainly occurs during the winter and spring seasons in China (16). It primarily attributed to the lower temperatures, creating conditions more conducive to the prolonged survival of the pathogens in the environment (22). However, the peaks of psittacosis infections were more commonly observed during the spring and summer seasons in the Netherlands (23). In Japan, approximately 49% of the cases occurred in May and July among the 115 reported cases from 2007 to 2016 (24). Similarly, recent surveillance data from European countries showed seasonal variations, with Austria, Denmark, Germany, Sweden, and the Netherlands reporting increased cases particularly marked since November–December 2023 (25). From a regional distribution perspective, the increasing number of reported cases of psittacosis in Guangdong Province were attributed to the improved diagnostic capabilities of subordinate cities. Currently, psittacosis is not classified as a notifiable infectious disease, leading to limited epidemiological research in China. Therefore, further in-depth research is necessary to investigate the seasonal patterns and regional clustering characteristics of psittacosis.

The actual incidence rate of psittacosis may be more severe, and this could be due to two main reasons. Firstly, psittacosis is not a notifiable infectious disease in China, thus the proportion of patients reported to NNDRS may be mostly serious patients. Besides, laboratory testing for psittacosis is not routinely performed in most hospitals. Many large hospitals in China need to send samples to third-party genetic testing companies for analysis, which may lead to delayed diagnosis, missed diagnosis, or misdiagnosis of psittacosis cases. Consequently, this increases the likelihood of cases progressing to pneumonia or becoming severe. International evidence supports this underestimation, with studies showing that reported cases represent only a fraction of actual infections. For instance, cases reported in the Belgian Statutory Infectious Disease Reporting System represent only 24% of laboratory-confirmed positive cases (26). Incorporating psittacosis into the surveillance system helps mitigate the worsening of the disease.

This study faced significant limitations regarding data completeness, with only 162 of 435 cases (37.2%) successfully followed up and included in the exposure history analysis. This substantial missing data represents a major limitation of our retrospective study design. Several factors contributed to this low follow-up rate: First, as psittacosis is not a notifiable infectious disease in China, there is no established comprehensive surveillance system requiring systematic follow-up of cases (27). Second, the retrospective nature of our study meant that many patients had been discharged and were difficult to contact for additional information collection. Third, some patients may have been reluctant to participate in follow-up interviews, particularly regarding their exposure to birds, which could be perceived as potentially problematic. Despite the incomplete follow-up data, our study successfully identified significant exposure patterns and risk factors among the cases with available information, providing valuable insights into psittacosis transmission dynamics. This study represents one of the first comprehensive investigations of psittacosis exposure patterns in Guangdong Province and provides important baseline data for future surveillance efforts. The findings from our available data consistently demonstrated clear associations between bird contact and infection, supporting the robustness of these key epidemiological relationships even with incomplete case ascertainment (28).

Contact with birds is the primary risk factor for *Chlamydia psittaci* infection. The main modes of exposure were through keeping and breeding these birds. Psittacosis has been found in more than 460 bird species, with the most common being parrots, pigeons, chickens, and ducks (29). In this study, the most frequently contacted bird species among the cases were chickens, parrots, and ducks, primarily sourced from food markets and bird markets. As people develop closer relationships with their pets, birds such as parrots have become important pets in some urban households. Birds that appear healthy but still shed *Chlamydia psittaci* are more likely to have their infection risk overlooked. Among the cases with a history of bird contact, few took personal protective measures, indicating a lack of public awareness about psittacosis. Therefore, it is necessary to strengthen public health education, particularly for high-risk poultry exposure workers and individuals who keep pet birds or poultry. When handling birds, they should use appropriate personal protective equipment, such as protective clothing, masks, and gloves (29).

Based on our findings and international experiences, targeted prevention strategies should be implemented for specific high-risk populations (30). Occupational groups including poultry workers, veterinarians, pet store employees, and bird breeders require specialized education programs about psittacosis risks and protective measures (11). It should emphasize proper use of personal protective equipment, including respiratory protection, gloves, and protective clothing when handling birds or cleaning bird areas. For older adult (≥50 years), who showed higher rates of severe disease in our study, enhanced diagnostic vigilance is recommended. Healthcare providers should maintain high clinical suspicion for psittacosis in older adults presenting with community-acquired pneumonia, especially those with any bird exposure history (31). Additionally, targeted diagnostic testing using advanced methods such as mNGS should be considered for older adult with underlying pulmonary disease, as early identification can prevent disease progression and improve outcomes (32).

Psittacosis is considered an underestimated infectious disease. Internationally, countries such as the United States, the Netherlands, and Belgium have classified psittacosis as a notifiable infectious disease (29). However, due to limitations in detection methods, difficulty in diagnosing mild cases, and underreporting, the reported number of psittacosis cases and the disease burden are significantly underestimated (23, 29). It was estimated that more than 1,500 symptomatic cases of psittacosis went undiagnosed from 2012 to 2014 in the Netherlands (15). Cases of psittacosis reported in the Belgian Statutory Infectious Disease Reporting System represent only 24% of the laboratory-confirmed positive cases (26). Due to the low level of attention given to psittacosis, its potential risks, such as the prognosis of infection in pregnant women and the outbreak of human-to-human transmission, are difficult to assess (3, 33–35). However, after the outbreak of COVID-19, the widespread application of metagenomics has increased the detection rate of psittacosis and has initially highlighted its severity. Recent outbreaks in European countries, including five deaths reported in 2024, underscore the importance of enhanced surveillance and rapid response capabilities (36). It suggests that psittacosis should warrant greater public health attention. It is recommended to consider classify psittacosis as a notifiable infectious disease to accurately monitor and assess its actual incidence and disease burden.

This survey, by analyzing the epidemic characteristics of psittacosis over the past 6 years, has identified risk factors for the disease and suggests that the burden of psittacosis may be underestimated. However, as a retrospective study, there is information bias in some cases. All psittacosis cases in this study were reported through an online reporting system, and since psittacosis is not currently a notifiable infectious disease, the cases reported are likely to be severe, leading to potential selection bias in the study sample. While missing follow-up data constitutes a limitation in our study, the existing data are reliable and sufficiently representative to reflect the overall population. Future prospective surveillance studies with mandatory reporting requirements would provide more comprehensive and unbiased data. To mitigate recall bias, we strengthened data validation and cross-referenced information from multiple sources, such as hospital records and laboratory reports, to verify diagnostic and exposure details.

## 5 Conclusion

In summary, this study analyzed 435 psittacosis cases reported in Guangdong Province from 2019 to 2024, demonstrating a continuous increasing trend in psittacosis incidence, with cases predominantly occurring during winter and spring seasons. Nansha District in Guangzhou, Boluo County in Huizhou, and Shunde District in Foshan were three distinct geographic hotspots. Demographic patterns showing higher risk in males and 50–79 age group, underlying diseases as a risk factor for pneumonia, and low protective measure usage among exposed individuals It underscored the importance of understanding the epidemiological characteristics and control measures of psittacosis.

## Data Availability

The original contributions presented in the study are included in the article/supplementary material, further inquiries can be directed to the corresponding authors.
